# Exploring consumers’ environmental ethical preferences in the context of unmanned aerial vehicle utilization for plant protection

**DOI:** 10.1038/s41598-023-30557-4

**Published:** 2023-03-06

**Authors:** Baoshu Wu, Jinlian Lu, Bo Zhou, Zhenjiang Song

**Affiliations:** 1 School of Business Administration, Jiangxi University of Finance and Economics, Nanchang, 330032 China; 2 College of Economics and Management, Jiangxi Agricultural University, Nanchang, 330045 China; 3 Rural Development Research Center of Jiangxi Province, Jiangxi Agricultural University, Nanchang, 330045 China; 4 Institute of Jiangxi Selenium-Rich Agricultural Research, Jiangxi Agricultural University, Nanchang, 330045 China

**Keywords:** Environmental social sciences, Environmental economics, Psychology and behaviour, Socioeconomic scenarios, Sustainability

## Abstract

The use of unmanned aerial vehicles (UAVs) has increased agricultural productivity, achieved food security, and eased the pressure associated with environmental degradation and population growth. However, consumer sentiment remains unclear. The results show that pressures regarding food safety, production safety, and ecological safety have different degrees of positive impact on perceived benefits but no significant impact on perceived barriers. They strongly influence both perceived benefits to the adoption of UAV plant protection agricultural products. Perceived benefits demonstrated a mediating role between the three safety pressures and the adoption of UAVs. Lay beliefs showed a positive moderating effect on perceived benefits and obstacles to the adoption of UAV-based plant protection products. Based on these findings, this paper concludes that consumers are developing new consumer ethics that integrate concepts of food safety, safe production, and regional environmental protection with their acceptance of new technology, which is directly dependent on the combined effect of environmental and consumer ethics. To promote sustainable development, policies must be further optimized on this original basis.

## Introduction

Climate change currently poses an ever-growing threat to food security globally^1,2^. Therefore, the agricultural sector urgently needs to transform production and consumption patterns to contribute to sustainable development^3,4^. Although the past half-century has seen governments worldwide adopt sustainable production^5^, sustainable consumption has not yet been widely accepted^6,7^. Meanwhile, the observation of practices in recent years demonstrates that the success of sustainable development depends on not only reducing greenhouse gases in the production system but also implementing sustainable development strategies among the production and consumption systems, informed by emergent environmental consumption ethics^8–10^.

In the context of production systems, sustainable global development requires the digital transformation of agriculture to promote sustainable production^11^. Using unmanned aerial vehicles (UAVs) is now a key factor for global agricultural production and economic stability^12,13^, and UAVs have become the most widely used digital production tool in China. Additionally, they are recognized for their capacity to replace the workforce, improve labor productivity, protect producers, and reduce ecological pollution^14^. Meanwhile, the digital technology of UAVs can promote the sustainable development of agriculture, a perspective widely accepted by researchers.

Plant protection using UAVs has been incorporated into the traceability systems of agricultural products, enabling the monitoring of plant growth^15^, disease patterns^16^, and crop-dusting^17^ and the accurate estimation of agricultural output^18^, allowing for the improved quality and efficiency of agricultural production. Meanwhile, the plant protection periods for double-cropping rice have been concentrated between May and June and between August and September, when most regions in China experience the hottest temperatures. Agricultural workers find it difficult to effectively spray pesticides by hand under strict protection in this weather, leading to frequent heatstroke and pesticide poisoning (due to abandoning gas masks at high temperatures)^19^. In this case, using UAVs can improve labor productivity and protect producers. Furthermore, plant protection using UAVs is based on quantification and precision, enabling the strict control of the dosage and spraying range of a pesticide, reducing contamination of the surrounding areas, and improving environmental benefits^20^. This effort requires investigating the sustainable production problem in terms of three dimensions: food safety, production safety, and ecological safety.

In the context of the consumer system, the concerns about sustainable production relate to the environmental consumption ethics that has arisen in response to the global climate crisis^21–23^. Because the consumption patterns of China’s urban residents have moved from subsistence- to comfort-oriented^24–27^, consumers respond positively to sustainable development initiatives. However, sustainable development requires the joint efforts of the production and consumption systems. The production system has seen the introduction of digital technology to promote food safety, production safety, and ecological safety. In addition, the consumer system has encouraged living in harmony by conforming to environmental consumption ethics linking society, nature, and individuals. Although scholars have studied the consumer adoption of green agricultural products, the research is lacking regarding consumer response in terms of environmental consumption ethics and its internal driving mechanism in the context of technological progress. Scholars need to study the transformation of consumption intentions and behavior from systematic and multi-level perspectives and in terms of interrelationships^28^. In other words, rather than simply targeting individual consumers, potential policy solutions need to be systematically proposed. Therefore, this paper integrates qualitative research and experimental design organically, adopting the paradigm of the *triple rationality of consumption* (rationality of food, production, and ecological safety) to supplement the current trends in ethical economics usefully. Meanwhile, this research considers feasible solutions by comparing motivation and observable behavior.

This paper comprises six sections. First, this section introduces the research background, the current state of the research, and the extant research gaps. The second section presents the theoretical analysis and the construction of the theoretical model of “environmental consumption ethics.” Next, the research framework is described, proposing the hypothesis and constructing the research model based on pressure–state–response (PSR) theory. The fourth section details the research design, explaining the scale developed and the data collection process. Then, the data are analyzed, and the hypotheses are tested to obtain the research results. The final section concludes the research and presents recommendations based on the findings.

## Theoretical framework

Environmental ethics derives from people’s concerns for environmental issues and their thinking regarding relevant ethical norms^29^. Initially, environmental ethics brought non-human entities into the scope of ethical research and adjusted the relationship between humans and nature via morality^30^, ultimately realizing ecological civilization as a mode of sustainable development^31^. Therefore, sustainable development represents a research objective of environmental ethics.

The current research on environmental ethics aims to achieve environmental protection goals by regulating human behavior^32–34^. In the production context, environmental ethics has greater demands around productive behavior, including pesticide reduction, pollutant control, and technical efficiency improvement. Meanwhile, agricultural products from processes that meet the requirements of environmental ethics can be identified as environmentally ethical products. In the consumption context, the intersection between environmental ethics and general moral norms produces consumer ethics^35^. Consumer ethics describes the moral concepts people use in the consumption mode and consumption environment. It indicates the value judgments and moral evaluations associated with social consumption behavior and focuses on the relationships between society, nature, and individuals. These relationships correspond to the ecological, social, and individual dimensions^36^. The ecological dimension emphasizes the establishment of a relationship between humans and nature that is egalitarian and enabled by harmonious symbiosis. The social dimension pertains to the obligation of humans to maintain the survival and development of the whole of human society, which includes decisions about consuming and consuming ethically. Finally, the individual dimension suggests that the essence of consumption concerns the satisfaction of needs, indicating that the purchase behavior of ethical consumers is affected by various moral constraints and the different needs of individuals. This includes the impact of products on the natural environment and natural resources^37^, the safety and survival of production workers^38^, and the food safety and nutritional health provided by the products^39^.

As intelligent machines, UAVs can standardize agricultural production, improve production efficiency, and reduce harm to workers and the environment. UAVs not only respond to environmental ethics at the production end but also contribute to the implementation of consumption ethics, enabling the realization of sustainable supply and promoting sustainable consumption.

Some aspects of environmental and consumption ethics are shared. Both advocate concern for ecology, the coordination of humans and nature, and sustainable development. However, they differ because environmental ethics mainly focuses on balancing human development and environmental protection. In contrast, consumer ethics focuses on the impact of human consumption behavior on the individual, other people, and the environment equally.

Meanwhile, to consider ethics as the bridge between consumption and production^40^ implies environmental and consumer ethics are the ethical norms regulating production and consumption behaviors, achieving sustainable supply and consumption, and delivering the holistic sustainable development of the environment, society, and individuals.

Scientific and technological progress can help solve the limitations associated with natural resources, eliminating the adverse effects of using natural resources and promoting sustainable development^41^. As a flagship tool for precision agriculture, UAVs can help agricultural systems operate at the right location, intensity, and time^42^, potentially achieving food safety, ecological safety, and production safety. Specifically, UAVs can effectively minimize pesticide residues to help achieve food safety, reduce the exposure of workers to pesticides, reduce production costs, improve yields, and increase worker income and farm profits. In addition, the implementation of precision fertilizer application irrigation can gradually improve the ecological environment, meet the goals of environmental and consumer ethics, and realize the symbiosis of sustainable supply and sustainable consumption. These advances have infinite potential for promoting the sustainable development process^43,44^.

**Figure 1:**
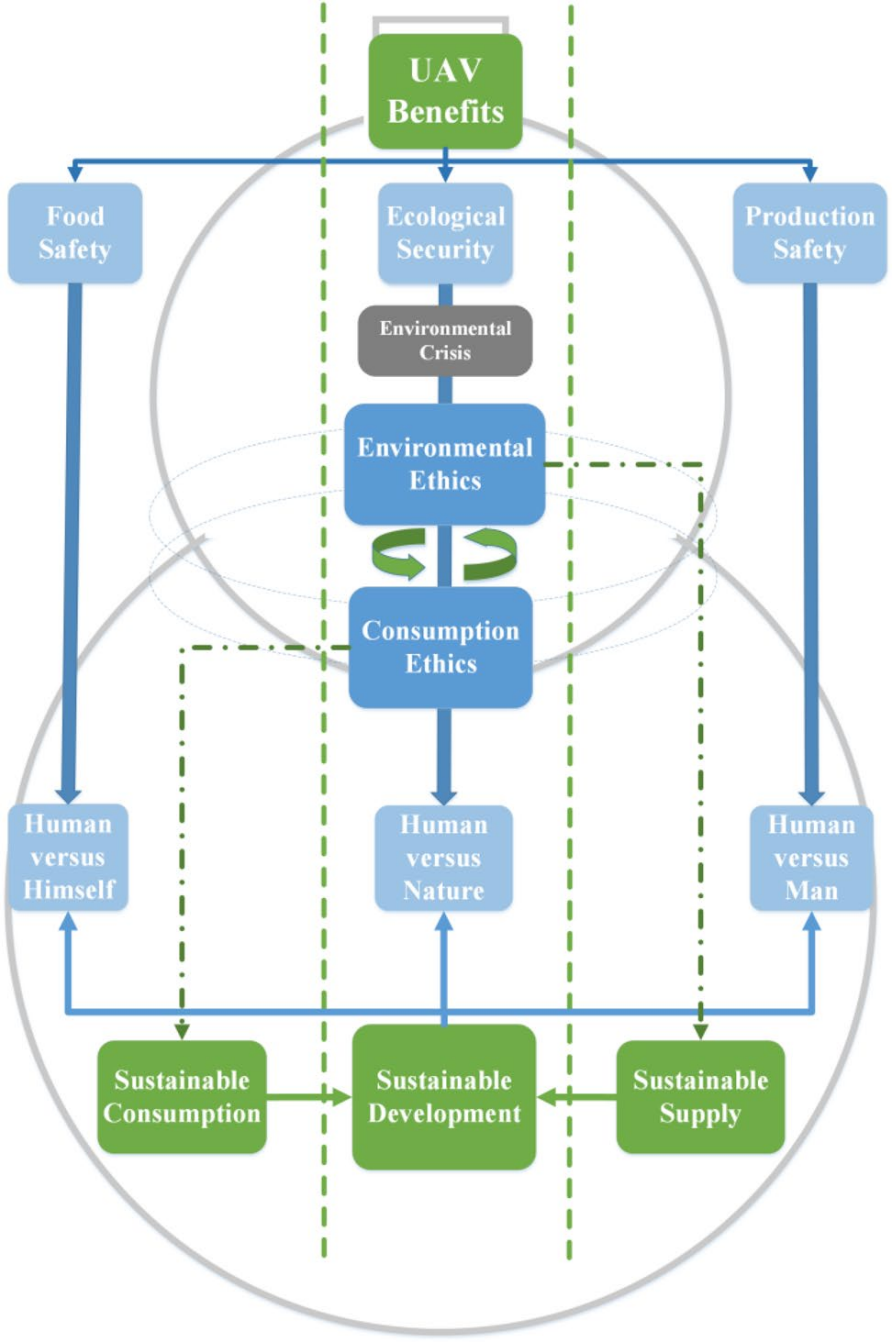
Model of environmental consumption ethics.

Accordingly, this paper constructs a theoretical model (Fig. 1) that features six logical components:

1. UAV benefits at the production end comprise food safety, ecological safety, and production safety.
2. The ecological-safety benefits of production-end UAVs address agriculture’s environmental crisis. The consumer ethics produced by the interaction between environmental ethics and general ethical theories at the production end focus on the relationship between humans and nature at the consumption end.
3. The production-safety benefits of production-end UAVs impact the relationship between individuals and others at the consumer end.
4. The food safety benefits of production-end UAVs impact the relationship between individuals at the consumer end.
5. Environmental ethics at the production end can provide sustainable supply, with consumer ethics at the consumption end enabling sustainable consumption. Sustainable supply and sustainable consumption combine to contribute to sustainable development.
6. Sustainable development harmonizes the relationships between society, nature, and individuals.

## Conceptual framework and hypotheses development

**Figure 2:**
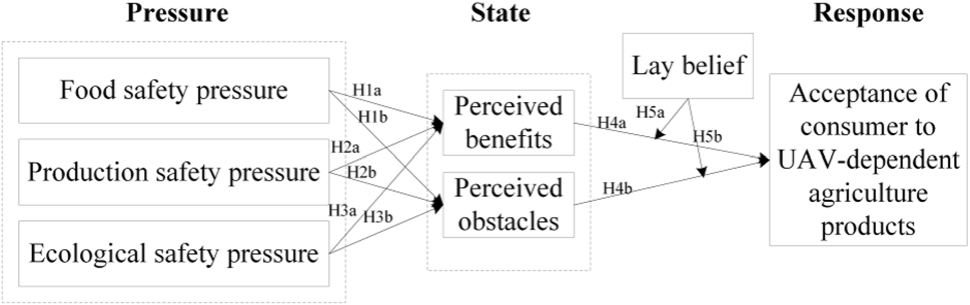
PSR research model.

Based on environmental consumption ethics^17^ and PSR theory^45^, this paper has constructed a research model describing the impacts of food-, production-, and ecological-safety pressures on perceived benefits and obstacles and the consequent acceptance of UAVs by consumers. Meanwhile, PSR theory has often been applied in the field of environmental economics to reveal the interactions between humans and the environment. Lay beliefs have been used to moderate the effects of perceived benefits and obstacles on consumer acceptance. Hence, based on the theoretical model analysis framework (Fig. 2), the hypothesis that consumers will accept UAV-dependent agricultural products is proposed.

### Impact of food-safety pressure on perceived benefits and obstacles

Food safety closely relates to consumer health. The world has substantially solved the problem of hidden hunger since food safety problems caused by pesticide residue and excessive heavy metals have attracted increasing attention^46–48^. In addition, excessive concentrations and quantities of traditional manual pesticide spraying were common, and uneven local areas of large-scale mechanical spraying were evident with highly toxic pesticides. These practices have all caused additional serious issues, including inefficient pesticide use and soil and water pollution, undermining sustainable development.

Meanwhile, the rapid popularization of the internet and “we-media” (The term “we-media” refers to information posted online by the general public without verification) has enabled the constant transmission of this information to consumers, which encourages consumers to buy products with green, organic, and pollution-free safety certificates^49^. In this context, interest in new agricultural technologies that are clean, efficient, and accurate (e.g., UAVs) has increased significantly. UAVs can release natural enemies of pests or spray insecticides accurately^50^, allow for quantitative spraying to help agricultural products meet green production standards^51^, reduce physical damage to crops and soil^52^, and reduce consumer concerns about diseases caused by pesticide residues^53^. Given these positive effects that can enhance consumer confidence in food safety, the authors of this paper believe that consumers have a positive attitude toward agricultural products protected by UAVs. Thus, we state the following hypotheses:

#### H1a.

Food-safety pressure has a positive impact on the perceived benefits consumers associate with accepting UAV-dependent agricultural products.

#### H1b.

Food-safety pressure has a negative impact on the perceived obstacles consumers associate with accepting UAV-dependent agricultural products.

### Impact of production-safety pressure on perceived benefits and obstacles

Production safety relates to worker health and improved crop yields and productivity. As a labor-intensive industry, traditional agriculture relies heavily on physical labor, which poses various threats to the health of farm workers. For example, the artificial transplantation of rice seedlings during rice planting often causes falls and injuries, and the long-term exposure of the lower limbs to mud can cause various diseases^54^. In addition, restricted by education level and cognitive ability, farm workers ignore or fail to understand pesticide operation instructions, threatening their health and potentially causing poisoning or death^55,56^. The root causes of this phenomenon are the lack of effective control and the improper behavior resulting from the limited capacity of bounded-rationality actors. With the progress of technology, intelligent, accurate, and efficient production tools can effectively restrain the inappropriate behaviors of bounded-rationality actors. These tools not only free workers from physical labor but also reduce harm to workers by enabling more precise and less dangerous pesticide spraying operations.

Furthermore, studies have shown that UAVs can improve the technical efficiency of agricultural production, and precision pesticide spraying can improve crop yields^57^. Moreover, consumers pay attention to not only the appearance, taste, and nutrition of agricultural products but also the labor process and health of workers at the production end. This represents a desire for enhanced levels of social welfare and the protection of worker health, which inclines consumers to pay higher prices for agricultural products manufactured according to higher moral standards^58^. Based on this ethical thinking, this paper proposes the following hypotheses:

#### H2a.

Production-safety pressure has a positive impact on the perceived benefits consumers associate with accepting UAV-dependent agricultural products.

#### H2b.

Production-safety pressure has a negative impact on the perceived obstacles consumers associate with accepting UAV-dependent agricultural products.

### Impact of ecological-safety pressure on perceived benefits and obstacles

Ecological safety closely relates to environmental ethics and sustainable development goals per global consensus, with the fundamental goal of protecting the planet and ensuring human well-being^59^. The essential purpose of environmental ethics is to improve the ecological environment and reduce the pressures associated with ecological insecurity. When linking supply and marketing, the environmental ethics, moral obligation, and green attitude of consumers combine to generate a consumption ethics that pushes consumer behavior toward greener consumption and the support of green production^60,61^. At the agricultural production end, the irrational use of pesticides and other chemical agents contributes to soil and water pollution, resource waste, and reduced biodiversity. At the consumption end, consumers are also affected by soil and water pollution, including the negative impacts of species reductions and extinctions. Intelligent, accurate, and efficient production tools, such as UAVs, can effectively control soil and water pollution during pesticide application, improving water-use efficiency and reducing disturbances to biodiversity. This can weaken the negative impacts of agricultural production on the ecological environment and improve the overall performance of agricultural production in terms of sustainable development. Therefore, UAVs in agriculture can positively impact the ecosystem and respond to the urgent consumer desire to improve the environments surrounding agricultural production areas. Accordingly, this paper proposes the following hypotheses:

#### H3a.

Ecological-safety pressure has a positive impact on the perceived benefits consumers associate with accepting UAV-dependent agricultural products.

#### H3b.

Ecological-safety pressure has a negative impact on the perceived obstacles consumers associate with accepting UAV-dependent agricultural products.

### Influence of perceived benefits and obstacles on acceptance behavior

A strong positive relationship exists between perceived benefits and acceptance, and a strong negative relationship occurs between perceived obstacles and acceptance^62–64^. Perceived benefits represent the overall evaluation of consumer perceptions of the quality and contribution of products^65^ and strongly influence those who want to purchase a given product^66^. Perceived benefits are also considered a significant factor for predicting individual behavior^67^, with higher perceived values associated with higher levels of satisfaction and loyalty and a greater willingness to accept new products^68^. Therefore, perceived benefits––as an essential mediating factor––link product characteristics with the behavioral responses of consumers, helping to reveal the specific mechanism of these relationships^69^.

Meanwhile, in the context of this research, perceived obstacles contribute to consumer tendencies to resist new technologies and associated products. For example, consumers may consider a given new technology to be pseudoscience or infeasible for implementation because of some imperfection^70^. Therefore, consumer recognition or acceptance of the value of UAV-dependent agriculture is reflected in their subjective evaluation of the related agricultural products. When such products meet consumer needs and conform to their ethical norms and other relevant values, they are more likely to be accepted. In contrast, when consumers perceive this acceptance as risky, they tend not to accept UAV-dependent agricultural products. Accordingly, this paper proposes the following hypotheses:

#### H4a.

Perceived benefits have a positive impact on the consumer acceptance of UAV-dependent agricultural products.

#### H4b.

Perceived benefits have a negative impact on the consumer acceptance of UAV-dependent agricultural products.

### Influence of lay beliefs on acceptance behavior

Lay beliefs refer to the judgment and choices of people without professional knowledge^71^. The most significant difference between experts and ordinary consumers is that experts can understand product attributes that non-experts cannot understand^72^. However, experts comprise a minority of consumers, and most consumers are non-experts. Therefore, most consumers make purchase decisions according to incomplete information based on product labels or mobile phone searches^73^. Although this decision-making process lacks a scientific basis, it is rational for ordinary consumers. Using UAVs in the agricultural production context remains novel in China, and everyday consumers have a minimal understanding of the technology. Therefore, this paper considers whether lay beliefs moderate the influence of perceived benefits and barriers on adoption by proposing the following hypotheses:

#### H5a.

Lay beliefs significantly moderate the impact of perceived benefits on consumer acceptance of UAV-protected agricultural products.

#### H5b.

Lay beliefs significantly moderate the impact of perceived obstacles on consumer acceptance of UAV-protected agricultural products.

## Methodology

### Construct designs

The measures in this paper derive from existing research, with modifications made to accommodate the current research context to arrive at the initial scale. Subsequently, the researcher invited 50 consumers to participate in pre-testing. According to their feedback, the wording of the initial scale was improved, resulting in a formal questionnaire comprising 25 measures. The questionnaire was measured using a Likert scale, where responses from 1 to 5 represent “strongly disagree,” “disagree,” “uncertain,” “agree,” and “strongly agree.” The finalized scales and sources are listed in Table 1.

**Table 1:** Constructs and sources.

Constructs	Items		References
Food-safety pressure (FSP)	FSP1	UAVs can target natural enemies of pests or spray insecticides with precision	April et al. 2019; Matthews^51^; Meng et al.^52^; Maik et al. 2019
	FSP2	UAVs’ quantitative spraying of pesticides can be more consistent with the green production standards of agricultural products	
	FSP3	UAV operations reduce damage to crops and soil, reducing adverse effects	
	FSP4	UAVs can reduce agricultural residue and ease consumer concerns about long-term chronic diseases, liver diseases, cancers, malformations, and genetic mutations caused by pesticide residue	
Production-safety pressure (PSP)	PSP1	UAVs can lighten labor and create a more relaxed and happier environment for workers in production activities	Rahman et al. 2021; Zheng et al. 2018; Mahroof et al. 2021
	PSP2	UAV operations are safer, and they can effectively reduce the harmful effects of pesticides on workers	
	PSP3	UAV operations are safe and efficient, increasing crop yields	
Ecological-safety pressure (ESP)	ESP1	UAV operations are conducive to reduced pesticide usage, water usage, and soil pollution	Mogili et al. 2018; Wang et al.^62^; Lan et al. 2021
	ESP2	UAV operations can save water and reduce water waste	
	ESP3	UAVs operate with precision, killing pests without harming beneficial creatures and maintaining biodiversity	
Perceived benefits (PB)	PB1	I think the mode and behavior of production and consumption should be conducive to environmental protection	Wang et al. 2021; Lan et al. 2019
	PB2	I learned that the use of UAVs may improve food safety	
	PB3	I learned that the use of UAVs may reduce harm to workers	
	PB4	I learned that the use of UAVs may improve the ecological environment	
	PB5	I learned that the use of UAVs may help produce agricultural products more efficiently	
Perceived obstacles (PO)	PO1	I heard that UAV technology is not perfect yet and the liquid drift phenomenon happens easily, which leads to environmental pollution and damage to surrounding organisms	Zheng 2021; Mahroof et al. 2021; Francisco et al. 2021
	PO2	I have heard that UAVs require professional maintenance and operation, which are difficult for ordinary farmers	
	PO3	I have heard that some problems with battery life and wind protection exist and UAVs are still in their infancy, meaning that large-scale applications may be difficult	
	PO4	I have heard that drones are expensive to operate and require professional evaluations to determine whether they are profitable	
	PO5	I have heard that many places are no-fly zones, meaning significant red tape is associated with reporting when a UAV launches	
Lay belief (LB)	LB1	I am not an expert on UAV-based agricultural applications, and I think UAVs can be suitable for agricultural production	Sydney et al. 2020
	LB2	I am not an expert on UAV-based agricultural applications, and I think UAVs may not be suitable for agricultural production	
Acceptance (AC)	AC1	I would like to buy UAV-dependent agricultural products immediately	Lin et al. 2019
	AC2	I would like to buy UAV-dependent agricultural products	
	AC3	I would like to buy UAV-dependent agricultural products	
	AC4	I will recommend buying UAV-dependent agricultural products to my friends and relatives	
	AC5	I will give UAV-dependent agricultural products to my friends and relatives	

### Data collection

This paper’s data were collected from August to September 2021 and obtained via online questionnaires sent using Wenjuanxing (Wenjuanxing is widely used in SCI1 area journals, including *PLoS One* (Song C., Liu W., Liu Z. et al. User abnormal behavior recommendation via multilayer network. PLoS ONE, 2019, 14(12):e0224684.), *Journal of Medical Internet Research* (Li M., Liu L., Yang Y. et al. Psychological Impact of Health Risk Communication and Social Media on College Students During the COVID-19 Pandemic: Cross-sectional Study. Journal of Medical Internet Research, 22(11):e20656.), and *Frontiers in Pharmacology* (Guo C., Hu B., Guo C. et al. A Survey of Pharmacogenomics Testing Among Physicians, Pharmacists, and Researchers From China. Frontiers in Pharmacology, 2021, 12.).), one of China’s most popular online survey platforms. The questionnaires were completed after respondents received an explanation of the purpose of the research and watched a short video featuring operational UAVs in practice. Furthermore, to improve the validity of responses, we paid each participant 5 RMB in exchange for completing the survey. A total of 300 questionnaires were randomly distributed, and 288 valid questionnaires were obtained after eliminating those featuring the same answer for every question. Most respondents were characterized by a university-level education and farming experience (56.9%). Accordingly, many exhibited some understanding of agricultural production, cared about food safety and ecological safety, and easily understood the questionnaire contents. Therefore, this paper’s research data can be considered relatively representative. The demographic characteristics of the study sample are shown in Table 2.

**Table 2:** Sample characteristics (N = 288).

Item	Category	Number	Percentage	Item	Category	Number	Percentage
Gender	Male	139	48.3	Area	City	150	52.1
	Female	149	51.7		Countryside	138	47.9
Education	Elementary school or below	6	2.1	Monthly household income	RMB 4000 or less	72	25
	Junior high school	30	10.4		RMB 4000–6000	71	24.7
	High school/specialized secondary schools	48	16.7		RMB 6000–8000	44	15.3
	Undergraduate studies/junior college	169	58.7		RMB 8000–10,000	36	12.5
	Postgraduate and above	35	12.2		above	65	22.6
Job	Civil servants/public institutions	37	12.8	Have you ever had farming experience?	Yes	164	56.9
	Entrepreneurs	7	2.4		No	124	43.1
	Enterprise staff	36	12.5	–	–	–	–
	Individual business	25	8.7	–	–	–	–
	Farmers	40	13.9	Have you ever used a UAV?	Yes	35	12.2
	Students	80	27.8		No	253	87.8
	Other	63	21.9	–	–	–	–

(1) The ratio of males to females in the sample is 1:1.07, which is balanced.

(2) The educational level structure of the interviewees was reasonable, with 2.1% indicating elementary school education or below, 10.4% indicating junior high school education, 16.7% indicating high school/specialized secondary school education, 58.7% indicating undergraduate studies/junior college education, and 12.2% indicating postgraduate or above.

(3) The interviewees have a reasonable occupation structure, with civil servants/public institutions representing 12.80% of respondents, entrepreneurs representing 2.40% of respondents, enterprise employees representing 12.50% of respondents, self-employed business people representing 8.70% of respondents, farmers representing 13.90% of respondents, and students representing 27.80% of respondents.

(4) Urban respondents accounted for 52.10% of all participants, and rural respondents accounted for 47.90%. This regional distribution structure was reasonable.

(5) The income structure of respondents was reasonable. The income group earning less than RMB 4,000 accounted for 25.00% of respondents, the income group earning RMB 4,000–6,000 accounted for 24.70% of respondents, the income group earning RMB 6,000–8,000 accounted for 15.30% of respondents, the income group earning RMB 8,000–10,000 accounted for 12.50% of respondents, and the income group earning more than RMB 10,000 accounted for 22.60% of respondents.

(6) A total of 56.90% of respondents had farming experience, and 43.10% had no farming experience. The structure of the farming experience was reasonable.

(7) A total of 29.51% of participants indicated that they had used a UAV previously. This proportion was unusually high.

### Ethical statement

There are no ethical issues involved in this study. And questionnaire has been approved by Academic Committee of Institute of New Rural Development, Jiangxi Agricultural University, which could perform the function of ethics committee.

### Relevant guidelines and regulations

All methods were carried out in accordance with relevant guidelines and regulations.

### Informed consent

Informed consent was obtained from all subjects.

## Data analysis and hypothesis testing

### Common method bias

First, this paper employed SPSS 26.0 to perform principal component analysis and use the maximum variance rotation method for factor analysis, with the measures of the seven structural variables merged into one variable. According to the results, the KMO (Kaiser–Meyer–Olkin) value was 0.902 (exceeding 0.8), Bartlett’s test value was 5279.914, the DF (Degree of Freedom) value was 351, and the statistical significance was 0.000, indicating that the data used in this paper were suitable for the factor analysis. Finally, Harman’s single-factor test was performed on the sample data. Five factors were extracted from the factor analysis results; the variance contribution rate of the first factor was 34.692%, and no single factor accounted for most of the co-variance. Therefore, no serious common method bias was present, making it feasible to analyze the relationships among this paper’s variables.

### Reliability tests

Smart PLS 3.0 was used to test the data for variables (i.e. food-safety pressure, production-safety pressure, ecological-safety pressure, perceived benefits, perceived obstacles, and acceptance). The load values of all measures ranged from 0.735 to 0.922, exceeding the critical value of 0.600. Meanwhile, Cronbach’s ɑ value was used to test the data’s reliability, and these values ranged from 0.803 to 0.915, which exceeded the critical value of 0.600, indicating acceptable reliability. The CR (Composite Reliability) values of all factors ranged from 0.884 to 0.936, exceeding the critical value of 0.700, indicating that the scale was reliable (see Table 3).

**Table 3:** Reliability, convergence validity, and collinearity testing (N = 288).

Construct	Item	Mean	SD	Factor Loading	Cronbach’s ɑ	CR	AVE
Food-safety pressure	FSP1	3.997	0.874	0.814	0.849	0.898	0.689
	FSP2	4.010	0.873	0.841			
	FSP3	3.983	0.885	0.837			
	FSP4	3.774	1.013	0.828			
Production-safety pressure	PSP1	4.351	0.682	0.834	0.803	0.884	0.717
	PSP2	4.351	0.692	0.883			
	PSP3	4.076	0.835	0.823			
Ecological-safety pressure	ESP1	3.962	0.904	0.922	0.888	0.930	0.816
	ESP2	4.056	0.890	0.894			
	ESP3	3.788	0.995	0.895			
Perceived benefits	PE1	4.313	0.678	0.735	0.877	0.910	0.671
	PE2	3.986	0.826	0.883			
	PE3	4.257	0.665	0.765			
	PE4	4.042	0.804	0.850			
	PE5	4.149	0.739	0.851			
Perceived barriers	PO1	3.368	0.893	0.845	0.915	0.936	0.745
	PO2	3.795	0.935	0.867			
	PO3	3.590	0.966	0.887			
	PO4	3.601	0.972	0.892			
	PO5	3.559	0.943	0.822			
Acceptance	AC1	4.125	0.661	0.855	0.911	0.934	0.739
	AC2	2.559	1.182	0.899			
	AC3	3.080	0.997	0.901			
	AC4	3.292	0.980	0.836			
	AC5	3.368	0.935	0.801			

Data sources: Calculation and arrangement of this paper.

### Validity analysis

Validity reflects the accuracy of the data. The validity test results show that the average variance extracted (AVE) of all factors exceeded 0.500, and the loading of all factors exceeded 0.500, indicating good convergence validity for the scale measures (see Table 3).

The square root of the AVE of each factor exceeded the correlation coefficients between that factor and the other factors, indicating that scale items feature good discriminant validity (see Table 4).

**Table 4:** Discriminant validity test.

Construct	FSP	PSP	ESP	PBE	PBA	AC
FSP	**0.830**					
PSP	0.650**	**0.847**				
ESP	0.725**	0.603**	**0.903**			
PB	0.648**	0.682**	0.690**	**0.819**		
PO	− 0.028	0.020	0.028	0.041	**0.863**	
AC	0.415**	0.300**	0.396**	0.405**	0.217**	**0.860**

The boldface values on the diagonal are the AVE square-root values, and the other values represent between-plane correlation coefficients.

N = 228; ** p < 0. 01 for the two-tailed test.

Data sources: Calculation and arrangement of this paper.

### Hypothesis testing

**Figure 3:**
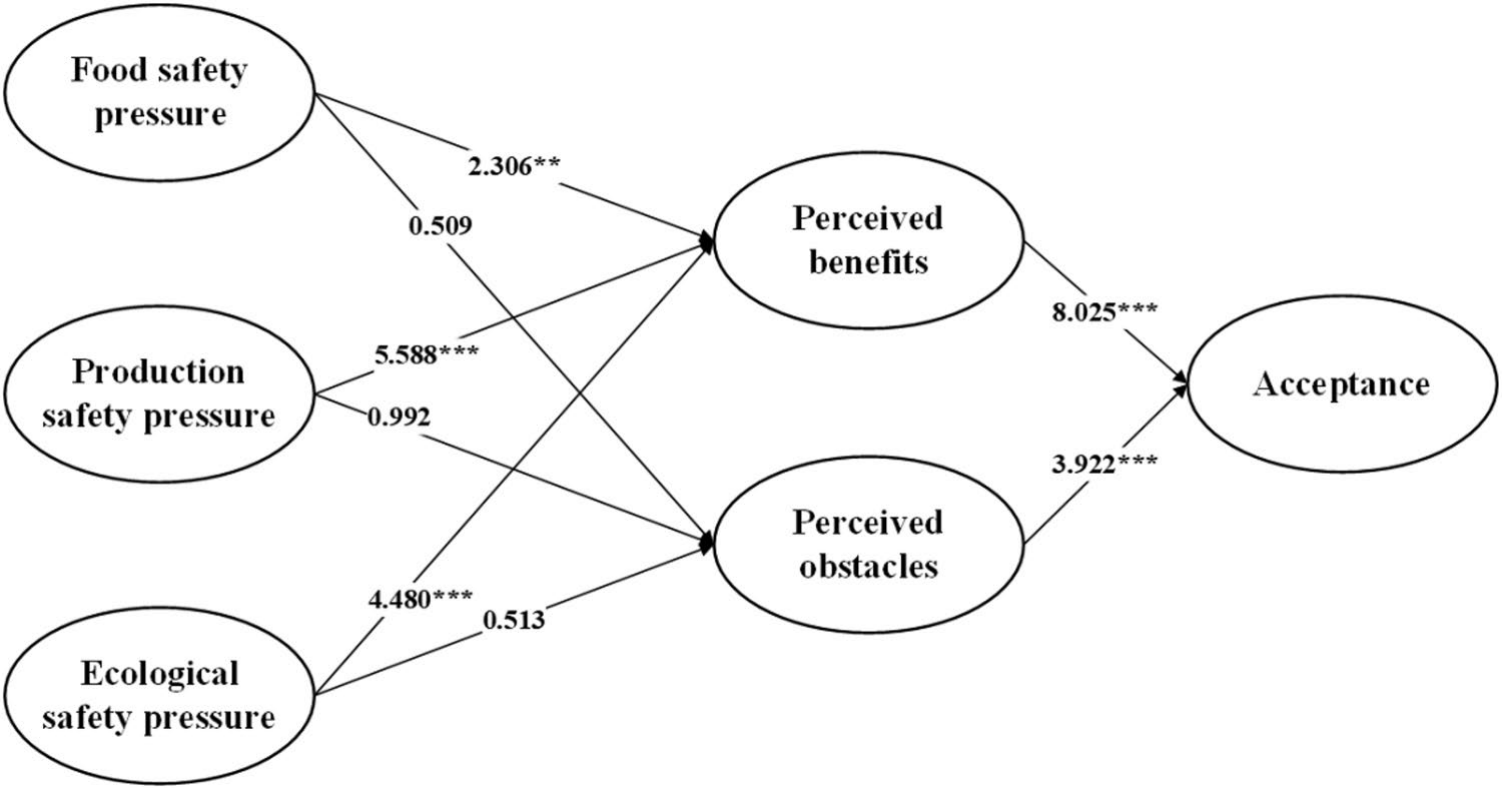
Results diagram. Note: *** *p* < 0. 001, ** *p* < 0. 01, * *p* < 0. 05. Data sources: Calculation and arrangement of this paper.

This paper used SmartPLS3.0 to build a global structural equation model to test the proposed hypotheses and explore the relationships among FSP, PSP, ESP, PBE, PBA, and AC and the possible operational mechanism. Figure 3 shows that FSP had a positive effect on perceived benefits (β = 2.306, *p* < 0.001), supporting H1a, but it had no significant effect on perceived obstacles (β = 0.509, *p* > 0.05), failing to support H1b. PSP had a positive effect on perceived benefits (β = 5.588, *p* < 0.001), supporting H2a, but it had no significant effect on perceived obstacles (β = 0.992, *p* > 0.05), failing to support H2b. ESP had a positive effect on perceived benefits (β = 4.480, *p* < 0.001), supporting H3a, but it had no significant effect on perceived obstacles (β = 0.513, *p* > 0.05), failing to support H3b. PB (β = 8.025, *p* < 0.001) and PO (β = 3.922, *p* < 0.001) both exhibited significant effects on purchase intention, supporting H4a and H4b.

### Mediation analyses

This paper adopted Model 4 in SPSS PROCESS3.3––a simple mediation model prepared by Hayes (2012)^74^––to test the mediating effects of the relationships between FSP, PSP, ESP, and AC.

The results demonstrate that both FSP and ESP had significant predictive effects on AC (*t* = 7.708, *p* < 0.001; *t* = 7.298, *p* < 0.001) (Tables 5 and 6). When the intermediate variables were added, the direct prediction effect of FSP and ESP on AC remained significant (*t* = 3.790, *p* < 0.001; *t* = 3.031, *p* < 0.001; respectively). The upper and lower bounds of the bootstrap 95% confidence interval of the direct and mediating effects of FSP and ESP on AC did not contain 0 (Table 5), indicating that FSP and ESP can predict purchase behavior directly and via the mediating effect of PB. The influence of FSP and ESP on AC via the direct and mediation effects of PB recorded a bootstrap 95% confidence interval with the lower limit not containing 0 (Table 5), suggesting that FSP and ESP can predict purchase behavior directly and via the PB of mediation role purchase behavior. The direct effect (0.277) and the intermediary effect (0.160) of the relationship between FSP and AC represent 63.40% and 36.63%, respectively, of the total effect (0.4371). The direct effect (0.212) and intermediary effect (0.164) of the relationship between ESP and AC represent 56.34 and 43.66%, respectively, of the total effect (0.3761).

**Table 5:** Mediation model testing of perceived benefits (N = 288).

AC	Model 1		AC	Model 2		AC	Model 3	
	β	t		β	t		β	t
FSP	0.437	7.708***	FSP	0.522	14.371***	FSP	0.277	3.790***
PB			PB			PB	0.306	3.381***
R^2^	0.172		R^2^	0.419		R^2^	0.204	
F	59.405***		F	206.514***		F	36.503***	

AC	Model 4		AC	Model 5		AC	Model 6	
	β	t		β	t		β	t
PSP	0.384	5.322***	PSP	0.6677	15.752***	PSP	0.058	0.614
PB			PB			PB	0.488	5.053***
R^2^	0.090		R^2^	0.4645		R^2^	0.165	
F	28.320***		F	248.1096***		F	28.139***	

AC	Model 7		AC	Model 8		AC	Model 9	
	β	t		β	t		β	t
ESP	0.376	7.298***	ESP	0.501	16.125***	ESP	0.212	3.031**
PB			PB			PB	0.328	3.403***
R^2^	0.157		R^2^	0.476		R^2^	0.190	
F	53.263***		F	260.011***		F	33.406***	

In Model 1, food-safety pressure predicts acceptance. In Model 2, food-safety pressure predicts perceived benefits. In Model 3, food-safety pressure and perceived benefits predict acceptance. In Model 4, production-safety pressure predicts acceptance. In Model 5, production-safety pressure predicts perceived benefits. In Model 6, production-safety pressure and perceived benefits predict acceptance. In Model 7, ecological-security pressure predicts acceptance. In Model 8, ecological-security pressure predicts perceived benefits. In Model 9, ecological-security pressures and perceived benefits predict acceptance.

Data sources: Calculation and arrangement of this paper.

*** p < 0. 001, ** p < 0. 01, * p < 0. 05.

**Table 6:** Total, direct, and intermediate effects.

FSP-PBE-AC	Effect	BootSE	BootLLCI	BootULCI	Proportion
Total effect	0.437	0.065	0.311	0.570	
Direct effect	0.277	0.081	0.126	0.448	63.40%
Intermediate effect	0.160	0.048	0.059	0.249	36.63%
PSP-PBE-AC					
Total effect	0.384	0.076	0.236	0.533	
Direct effect	0.058	0.099	− 0.127	0.270	–
Intermediate effect	0.326	0.070	0.196	0.468	
ESP-PBE-AC					
Total effect	0.376	0.062	0.259	0.503	
Direct effect	0.212	0.074	0.080	0.369	56.34%
Intermediate effect	0.164	0.047	0.069	0.258	43.66%

Data sources: Calculation and arrangement of this paper.

PSP demonstrated a significant predictive effect on AC (*t* = 5.322, *p* < 0.001). However, when the mediating variables were added, the direct effect of PSP on AC was not significant (*t* = 0.614, *p* > 0.05). Still, the mediating effect was significant (*t* = 5.053, *p* < 0.001), indicating that PB was a complete intermediary between PSP and AC.

These findings might be explained by consumers’ understanding of and focus on food and ecological safety to a greater extent than they do regarding specific UAV practices in agriculture.

### Moderate effects

To avoid multicollinearity among independent, dependent, and moderating variables, data for both independent and moderating variables were centrally processed in this paper. After the demographic variables were controlled, regression analysis was conducted on the independent variables, moderating variables, and interaction terms between the independent and moderating variables to predict the outcome variables, as listed in Table 7.

**Table 7:** Testing the moderating effect of lay beliefs (N = 288).

Construct	Item	AC				
		Model 1	Model 2	Model 3	Model 4	Model 5
Control variables	Constant	5.439***	5.483***	5.484***	5.246***	5.111***
	Gender	0.099	0.043	0.038	0.101	0.111
	Education	− 0.208***	− 0.201***	− 0.198***	− 0.18***	− 0.159***
	Monthly household income	− 0.048	− 0.047	− 0.04	− 0.052	− 0.034
	Area	− 0.201**	− 0.146	− 0.166*	− 0.209**	− 0.233**
	Job	− 0.032	− 0.042*	− 0.038*	− 0.019	− 0.014
	Farming or not	− 0.269***	− 0.305***	− 0.292***	− 0.265***	− 0.275***
	Using UAV or not	− 0.258*	− 0.241*	− 0.248*	− 0.232	− 0.234*
Independent variables	PB		0.548***	− 0.257***		
	PO				− 0.169***	− 0.229***
Moderating variables	LB			0.206***		0.216***
Product term	PB × LB			0.515**		
	PO × LB					0.124**
	VIF	1.026–1.497				
	F	5.012***	13.757***	12.274***	5.629***	6.539***
	R^2^	0.111	0.283	0.307	0.139	0.191
	△R^2^	0.089	0.262	0.282	0.114	0.162

Data sources: Calculation and arrangement of this paper.

*** p < 0. 001, ** p < 0. 01, * p < 0. 05.

According to Model 3, when the control variables LB, PB, and LB × PB are included in the regression equation, LB × PB significantly impacts PB (β = 0.515, *p* < 0. 05), indicating that LB positively regulates the relationship between PB and AC, supporting H5a. According to Model 5, when the regression equation considers the control variables LB, PO, and LB × PO, LB × PO significantly impacts PO (β = 0.124, *p* < 0. 05), suggesting that LB positively moderates the relationship between PO and AC, supporting H5b.

## Conclusions and implications

### Conclusions

The paper provides various theoretical contributions to the literature on sustainable consumption decisions by comprehensively considering the factors of climate, environment, and ecology from the perspective of consumer ethics.

First, this research promotes a comprehensive understanding of sustainable consumption decisions from academic and political perspectives at the climate, environment, and ethical levels. Most previous studies on sustainable consumption were based on the general situation. Individual studies focused on single factors affecting sustainable consumption decisions. However, those studies only summarized the influencing factors from specific perspectives, leading to a lack of in-depth discussion of sustainable consumption decision-making from a holistic perspective. Therefore, this paper analyzes the process by which consumers arrive at sustainable consumption decisions via the *triple rationality of consumption* of climate, environment, and ethics, enriching the current research system.

Second, the paper explores the influencing mechanism of sustainable consumption decisions within the *triple rationality of consumption paradigm*, which is based on ethical economics and an expansion of the current theoretical system. Recognizing that the research concerning the application of ethical economics in the context of consumer decision-making has not been applied in the context of *triple rationality of consumption*, this paper expands the depth and breadth of the research and application of ethical economics in the academic context.

Third, the effects of perceived benefits and barriers demonstrate the cognition on climate–environment–ecology and the ethical choice of consumers in the early stage of new technology popularization. Perceived benefits had significant mediation effects between *triple rationality of consumption* and acceptance, while perceived barriers had no significant mediation effects between them. This result indicates that the disadvantages of new technology have not been a major concern for consumers in the early stage of its popularization. In addition, technical barriers prevent consumers from forming an effective negative consensus. Therefore, the *triple rationality of consumption* can change with the development of technology and the deepening of consumer cognition, while the acceptance of consumers is dynamic.

Finally, this paper considers the influence of lay beliefs since most consumers are not experts. This novel research technique highlights the impacts of food safety, production safety, and ecological safety on consumer acceptance of agricultural products by integrating the effects of environmental and consumer ethics. Recognizing that consumer decisions are limited by the depth of consumer knowledge of technical issues, the consideration of non-expert beliefs reveals nonlinear characteristics of consumer decisions.

### Management implications

The issue of sustainable consumption decisions under the paradigm of the *triple rationality of consumption* remains in the early stages from a research perspective, with the concept of sustainable consumption yet to be fully adopted. For example, the separation of consumer sustainable consumption intentions and behaviors has been evident. Therefore, policy formulation should focus on stimulating sustainable consumption behavior more effectively to respond to the epochal topic of sustainable development. According to this study’s findings, several policy implications can be proposed.

First, governments must use diversified new media to strengthen the promotion of sustainable consumption for consumers and support sustainable development. For example, government departments should effectively publicize the production characteristics, nutritional quality status, and ecological impact of products to provide consumers with scientific evidence at the consumption stage. Meanwhile, government departments can inform consumers about sustainable consumption via the internet and authorized platforms. For example, public service announcements can be made using official mobile applications and platforms. Government departments should also generate positive publicity and guide food manufacturers in sales processes.

Second, basic information disclosure systems should be developed, strengthened, and maintained within the agriculture and food-production industries, which can support sustainable development at the system level. Objective and unbiased public disclosure is dominated by governments and supervised by consumers. This includes information concerning agricultural products, risk warnings, crisis repair, digital wildfire, packing, and consumer surveys. Furthermore, the guidance must be clear and reliable to prevent the dissemination of fraudulent information and promote the long-term development of sustainable consumption.

Finally, to promote research on ethical consumption and enhance the concept of sustainable consumption under the *triple rationality of consumption*, consumer demand for sustainable consumption can be aroused and addressed by ethical factors integrated into the brand name, packaging, advertising promotion, service promise, and marketing atmosphere. In addition, producers can enhance communications with consumers regarding ethical consumption via diverse approaches in the form of official mobile applications, WeChat accounts, websites, and press conferences, ultimately generating an atmosphere of sustainable consumption. In this context, the ecological visibility and reputation of enterprises can be improved to promote sustainable consumption intentions and the behavior of consumers within the *triple rationality of consumption* paradigm.

### Limitations and recommendations for future research

This paper applies a deductive method to study the internal logic of sustainable consumption via the theory of ethical economics under the *triple rationality of consumption*. Furthermore, the paper reviews the representative literature in various contexts to determine the mechanisms impacting sustainable consumption. However, the research methodology is relatively simple. Therefore, future research should use a variety of methods for a more comprehensive analysis of the factors that critically impact potential sustainable consumption.

Second, the paper employs cross-sectional data. Future research should explore changes in sustainable consumption behavior. Accordingly, the research group plans to build panel data based on this paper to improve the empirical usefulness of the data.

## Data Availability

Correspondence and requests for materials should be addressed to Z.S. and B.Z.
